# Non-Isothermal Crystallisation Kinetics of Carbon Black- Graphene-Based Multimodal-Polyethylene Nanocomposites

**DOI:** 10.3390/nano9010110

**Published:** 2019-01-18

**Authors:** Ibrahim A. Ahmad, Hyun-Kyung Kim, Suleyman Deveci, R. Vasant Kumar

**Affiliations:** 1 Department of Materials Science and Metallurgy, University of Cambridge, 27 Charles Babbage Rd, Cambridge CB3 0FS, UK; iaiaa2@cam.ac.uk; 2 Gwangju Bio/Energy R&D Center, Korea Institute of Energy Research (KIER), 270-25 Samso-ro, Buk-gu, Gwangju 61003, Korea; 3 Innovation Centre, Borouge Pte Ltd., PO BOX 6951 Abu Dhabi, UAE; suleyman.deveci@borouge.com

**Keywords:** non-isothermal crystallisation kinetics, multi-modal polymer, graphene-based polymer nanocomposite, carbon black fillers.

## Abstract

The effect of carbon black (CB) and microwave-induced plasma graphene (g) on the crystallisation kinetics of the multimodal high-density polyethylene was studied under non-isothermal conditions. The non-isothermal crystallisation behaviour of the multimodal-high-density polyethylene (HDPE), containing up to 5 wt.% graphene, was compared with that of neat multimodal-HDPE and its carbon black based nanocomposites. The results suggested that the non-isothermal crystallisation behaviour of polyethylene (PE)-g nanocomposites relied significantly on both the graphene content and the cooling rate. The addition of graphene caused a change in the mechanism of the nucleation and the crystal growth of the multimodal-HDPE, while carbon black was shown to have little effect. Combined Avrami and Ozawa equations were shown to be effective in describing the non-isothermal crystallisation behaviour of the neat multimodal-HDPE and its nanocomposites. The mean activation energy barrier (ΔE), required for the transportation of the molecular chains from the melt state to the growing crystal surface, gradually diminished as the graphene content increased, which is attributable to the nucleating agent effect of graphene platelets. On the contrary, the synergistic effect resulting from the PE-CB nanocomposite decreased the Δ*E* of the neat multimodal-HDPE significantly at the lowest carbon black content.

## 1. Introduction

Multimodal high-density polyethylene (HDPE) is an engineered thermoplastic semi-crystalline polymer, which is widely used in films, pressure pipes, bottles, tubes and cables jacketing [1,2,3,4,5]. It is a hybrid of at least two distinct polyethylene components, wherein each constituent has different density and different molecular weight fractions [3,4]. This allows flexibility in engineering its microstructure to meet the desired balance of properties for concrete practical applications. Nevertheless, multimodal HDPE can be further improved, for example, with the addition of fillers or reinforcements, in order to overcome deficiencies in their mechanical or thermal properties [6,7,8,9,10,11,12,13,14,15,16,17,18]. However, the crystallisation kinetics of the final product can be influenced, for example, not only by the nature of the added fillers but also by changing the molar mass (M), the breadth of molecular weight distribution (MWD), and/or the mode of MWD [1,2,5,6,11,13,15,19,20,21,22,23,24]. The spherulitic growth rate is found to increase as molar mass reduces, while polymers with broader and/or bimodal MWD lead to an increased peak crystallisation temperature and overall crystallisation rate [1,2,5,6,11,15,19,20,21,22,23].

Graphene has recently emerged as a very promising breakthrough material in the field of polymer nanocomposites and has attracted considerable scientific interest [7]. A large number of studies have reported numerous property enhancements attained from the use of graphene in polymer nanocomposites, such as superior mechanical, thermal, gas barrier, electrical and flame retardant properties [7,8]. Carbon black is also widely used as a speciality additive in multimodal high-density polyethylene (HDPE) for enhancing properties such as wear resistance and jetness [9,10,12]. It is very well-known and widely used with multimodal-HDPE for pressure pipes and power cable jacketing applications [12]. The incorporation of such fillers or reinforcements may significantly affect the crystallisation behaviour of the multimodal-HDPE, as the matrix is a semi-crystalline polymer with both crystalline and amorphous regions [11]. A number of studies have reported on the effect of nanofillers, such as carbon nanotubes, diamond, graphite, talc, CaCO_3_, SiO_2_, TiO_2_, BaSO_4_, on the crystallisation kinetics of HDPE [11,13,24,25,26,27,28,29,30,31,32,33]. As an example, J-W. Huang et al. reported a decrease in the spherulite size and degree of crystallinity and an increase in peak crystallisation temperature and overall crystallisation rate with the addition of inorganic fillers [26]. J. Kim et al. observed that the introduction of the multiwalled carbon nanotubes to the HDPE hindered the chain ordering, thus increasing the time required to reach 50% of relative crystallinity, despite the increase in the onset and peak crystallisation temperatures [13]. The mechanism of the primary and secondary crystallisation processes has a profound impact on the arrangement, size and morphology of the crystallites, as well as the degree of crystallinity, thus affecting the mechanical and physical properties of the semi-crystalline polymers [1,5,11,26,34,35,36]. Therefore, a change in the crystallisation kinetics can alter properties such as modulus, barrier properties, post extrusion shrinkage, post-mould shrinkage and/or warpage, transparency or clarity, sagging, processing cycle times and heat resistance in HDPE [11]. Also, a knowledge of the conditions affecting the crystallisation kinetics is crucial for optimising the processing parameters. For example, inappropriate selection of the processing variables leads to many defects in injection-moulded artefacts such as warpage, dimensional instability, shrinkage and so forth. [30]. Moreover, a non-uniform wall thickness, shape distortion, ovality, and waviness are a result of cross-sectional and axial thermal-gradient variations in pipe extrusion [37]. 

Polymer crystallisation is a process that essentially involves two consecutive steps, namely the nucleation and growth of the crystal nucleus [6,19,34,35,36]. A fundamental kinetic model of the polymer crystallisation process, during both isothermal and non-isothermal conditions, provides the necessary framework for a better understanding of the influence of the nanofillers on the crystallisation behaviour and crystal morphology of the multimodal-HDPE. In general, crystallisation is often considered to take place under idealised isothermal conditions, which greatly simplifies the mathematical and thermodynamic analysis. However, it fails to account for the effect that the varying cooling rates and crystallisation temperatures have on the final properties of the polymer in real-world applications [2,11,13,24,26,28,29,34,35,36]. Therefore, it is of great practical significance to simulate industrial processes in order to study the crystallisation kinetics, as industrial processes often occur under non-isothermal conditions. In our previous study, we reported a novel method for the preparation of a high-performance polymer-graphene nanocomposite using a co-rotating intermeshing twin-screw extruder [8]. The project was conducted on one metric ton of a commercial polymer and more than one kilogram of graphene, on a semi-industrial scale extrusion system that can be scaled up to full industrial scale production. Accordingly, this article aims to study the effect of exfoliated microwave-induced plasma graphene on the crystallisation kinetics of the high molecular weight multimodal-HDPE, the polymer which undergoes crystallisation from the melt state during industrial processing and structural development, under non-isothermal conditions. A commercial carbon black/multimodal HDPE nanocomposite product is considered as a bench mark in this study, for the sake of comparison with new multimodal-HDPE, based on graphene. The incorporation of 0.1–5 wt.% carbon black or graphene nanofillers led the crystallisation kinetics of the multimodal-HDPE to behave differently in non-isothermal conditions. It is therefore important to understand the difference between the effect of these two nanomaterials on the crystallisation behaviour of the multimodal HDPE, as replacing the nanofiller, for example carbon black by graphene, requires a consequent optimisation of the processing parameters. The crystallisation kinetics were further explored, in an attempt to better understand the different roles, which the latter nanofillers play in the crystallisation processes of multimodal-HDPE. In this regard, a model proposed by T. Liu et al. on the basis of H. E. Kissinger, Avrami, and Ozawa equations were incorporated for better understanding of the crystallisation behaviours [11,13,24,25,29,38,39]. The H. E. Kissinger equation allows for the interpretation of the heterogeneous nucleation mechanism by estimating the activation energy barrier for nucleation, while T. Liu et al. model is utilised to fit the experimental results to allow better descriptions of non-isothermal kinetics by providing a relationship between the cooling rates and crystallisation temperature [38,39]. This study is of fundamental importance to the optimisation of processing variables, aiming to provide supplementary information about the non-isothermal crystallisation kinetics of carbon black and graphene-based multimodal-HDPE prepared by melt intercalation method. The results of this research provide greater insight into different processing factors, affecting the multimodal HDPE-graphene nanocomposite crystallisation performance and criterion for effectively producing the next generation of black multimodal-polyethylene compounds for use in high-pressure pipes, automotive and energy cable applications. This would, therefore, contribute to a better understanding of the relationship among processing-structure-property of the multimodal-HDPE and its nanocomposites. To the best of our knowledge, little research, if any, has studied and compared the crystallisation kinetics of multimodal-HDPE induced by a bottom-up graphene with a commercial multimodal-HDPE induced by a carbon black with an average primary particle size of 20 nm. 

## 2. Experimental

### 2.1. Materials

Unstabilised, high density polyethylene powders, produced with Ziegler Natta catalyst via a proprietary Borstar process (Borouge, United Arab Emirates), melt flow rate of 7.5 g/10 min (190 °C, 21.6 kg), Mw = 280 kg/mole, Mn = 8.49 kg/mole and Mw/Mn = 33 and a density of 950 kg/m^3^. Antioxidants masterbatch containing Irgafos 168 and Irganox 1010 were added to the polymers at 0.5 wt.% for optimum stabilisation during processing. Graphene powder was obtained from FVG Cambridge Nanosystems company (Cambridge, United Kingdom), with ≥96% carbon purity, bulk density of 0.0266 g/mL, flake thickness < 1.0 nm with an average lateral size range of 150–500 nm. Carbon Black powder was provided by Orion Engineered Carbons GmbH (Frankfurt am Main, Germany), with ≥92 cc/100g oil absorption number, ash content of 0.10%, Sulphur content of 0.10%, tint strength of 103%, average primary particle size of 20 nm and a density of 1.7–1.9 g/cm^3^ at 20 °C.

### 2.2. Nanocomposite Preparation

Both multimodal-HDPE/graphene (PE-g) and multimodal-HDPE/carbon black (PE-CB) were prepared via melt-intercalation method using Coperion ZSK 18 twin extruder having a screw diameter of 18 mm and a barrel length of 720 mm (L/D = 40). The screw rotation speed was 600 rpm, barrel temperature profile was in the range of 170–240 °C and feed rate was between 1–2 kg/h. For more information, the reader is directed to our previous work [8]. 

The nanofillers and a dry polyethylene powder were fed separately into the extruder via a spiral flow screw Brabender ISC-CM plus feeder. The nanofillers were fed at 0.1, 0.5, 1, 2 and 5 wt.% loadings. In order to prevent the polymer from degrading, an antioxidant masterbatch was simultaneously added through a side feeder, with the total loading of 0.5 wt.%. The extruded pellets were subsequently compression moulded to about 0.4 mm thickness, following ISO 293 under 5 MPa, at a temperature of 200 °C. This was undertaken via a compression moulding platen press (Dr. Collin P 400 M, Germany), for an overall programming cycle of 32 min at a heating and cooling rate of 15 °C/min. The specimens were successively conditioned at 23 ± 2 °C and 50 ± 5%, for at least 48 h, prior to being tested. 

### 2.3. Characterisation

**Polarised light microscopy (PLM)** analyses were conducted on a ZEISS Axio scope.A1 HAL 100/HBO 100, operated with an AxioCam MRc 5 camera and an AxioVision software. Film samples were sectioned to a thickness of 15 μm, using a fully automated rotary microtome Leica RM2265 (Leica microsystems, Wetzlar, Germany). Samples were cooled by DSC at 2.5 °C/min prior to being analysed under the polarised light. ImageJ software was used to calculate the mean particle size of the nanoparticle agglomerates and the %area fraction (200 × 200 µm^2^) upon the optical light microscopy and transmission electron microscopy (TEM) images. 

**Transmission electron microscopy (TEM)** was performed using Hitachi HT7700, at an accelerating voltage of 120 kV. Film samples were cryo-sectioned to a thickness of ~80 nm at −125 °C, using Leica EM UC7/FC7 Cryo-Ultra-microtome.

**Thermogravimetric analysis (TGA)** was carried out with Q500 TGA (TA instruments, New Castle, UK) with a heating rate of 10 °C/min from room temperature to 1000 °C in a nitrogen atmosphere. High-Resolution (Hi-Res)-Dynamic mode was performed with sensitivity of 2.00 and a resolution of 4 °C. 

**Rheological behaviour of the samples was** studied using stress-controlled rotational rheometer (Anton Paar Physica MCR 301 with CTD450 heating unit) at 190 °C under a nitrogen atmosphere. The compression moulded sample, weighing 1.5 g and with measurements of 25 mm in diameter and 1.5 mm in thickness was conditioned at 40 °C for 48 h. The sample was then placed onto a 25 mm parallel plate fixture and trimmed to a thickness of 1.2 mm by slowly lowering the upper plate. The dynamic frequency sweep was conducted from 500 to 0.0154 rad/s at 5% strain. The reason for starting from the maximum frequency was to avoid sample degradation under high temperature and low angular frequency speed. The polydispersity index (*PDI*) was calculated as follows [40]:

(1) $$PDI=\frac{100\;000}{G^{\prime }\left(\omega_{COP}\right)},\;\omega_{COP}=\omega \left(G^{\prime }=G^{\prime \prime }\right)$$

where *G*′ is the storage shear modulus, *G*” is the loss shear modulus, *ω* is the angular frequency and $\omega_{COP}$ is the crossover angular frequency point obtained from the intersection of storage modulus and loss modulus in a log-log scale of a frequency sweep test.

**Differential scanning calorimetry (DSC)** was performed in a N_2_ atmosphere (50 ± 5 mL/min), using approximately 5 mg of the sample, sealed in Al pans with TA Instruments Q2000. The samples were melted at 200 °C, then held for 10 min to eliminate the thermal history and subsequently cooled to 0 °C at cooling rates of 2.5, 5, 10 and 20 °C/min, respectively. The samples were again held at 0 °C for 10 min and subsequently heated to 180 °C at the same cooling rates as the prior cooling rate, in order to obtain the DSC exotherms. Heat flow, as a function of time and temperature, was recorded during both crystallisation and melting processes for consequent data analysis.

The peak crystallisation temperature (*T*_c_) was determined from the temperature at maximum heat flow of crystallisation peak. 

The following equation:

(2) $$\%X_{c}=\frac{\Delta H_{v}}{\left(1-W_{f}\right)\;{{\Delta H}_{v}}^{{}^{\circ}}}\times \;100\%$$

was used to calculate the degree of crystallinity (*X*_c_) of the polymers, where Δ*H_v_* is the enthalpy of fusion determined from the experiment, ΔH*_v_*° is the enthalpy of fusion of the 100% crystalline polymer (ΔH*_v_*° = 290 J/g) and *W_f_* is the weight fraction of the filler content in HDPE [2,34,35,36,41]. 

## 3. Results and Discussion

**Figure 1:**
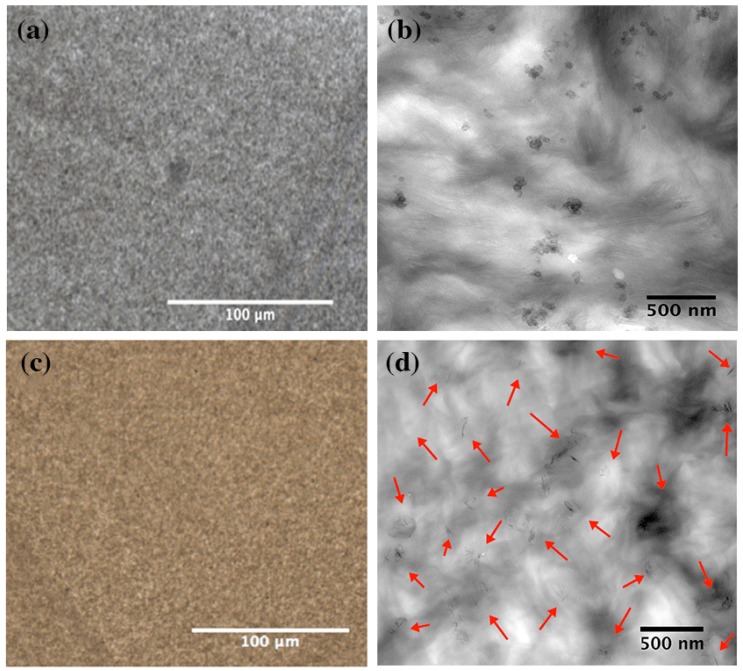
Light microscopy (**left**) and TEM images (**right**) show the dispersion and distribution of (**a**,**b**) carbon black and (**c**,**d**) graphene platelets within the multimodal-HDPE matrix. The red arrows show the distribution of graphene platelets throughout the polymer matrix. The TEM and light microscopy images were taken at 10k and 20×, respectively.

The dispersion and distribution of graphene and carbon black were assessed by TEM and light microscopy, as shown in Figure 1. More information about the details of our experimental protocol can be found in the literature [8]. Graphene monolayers are transparent under an optical microscope, opacity of 2.3 ± 0.1%, and the optical loss become greater in the wrinkled and overlapped samples [42,43]. L. J. Cote et al. found that the average light scattering from the wrinkled region is about 3.7 times that of the overlapped areas [43]. On the other hand, carbon black is composed of primary particles that are permanently fused together, through the covalent bonds, into an aggregate structure (see Figure S2) [44]. Each primary particle is made up of imperfect crystallites of turbostratic graphite structure, which are twisted into each other throughout the aggregates [44,45]. They are welded in the aggregate and are neither discrete nor do they have physical boundaries between them. Due to the production and storage conditions, the aggregates join together into agglomerates by van der Waal’s interactions [45]. The mean particle size of the carbon black agglomerates and the %area fraction (200 × 200 µm^2^) were around 0.9 µm^2^ and 0.4 respectively, as shown in Figure 1 and supplementary Figure S1. However, the mean particle size of the detected graphene agglomerates was 0.95 µm^2^ and the %area fraction was less than 0.006. The %area fraction and mean particle size were calculated based on TEM and light microscopy analysis and graphene particles of less than 0.05 µm^2^ or 500 nm were excluded from the calculations. A decrease in the %area fraction means a better distribution and fewer agglomerates. The figure was higher with carbon black as the fused aggregate sizes are commonly bigger than graphene monolayers. As shown from the TEM images in Figure 1, graphene platelets were thoroughly dispersed and distributed within the polymer matrix. However, carbon black images showed good distribution with different agglomerate sizes, though containing small sizes, which is normal in carbon black-based polymer nanocomposites [12,44,45]. Note that PE-CBs were used as a benchmark in this study in the interest of comparison. 

**Figure 2:**
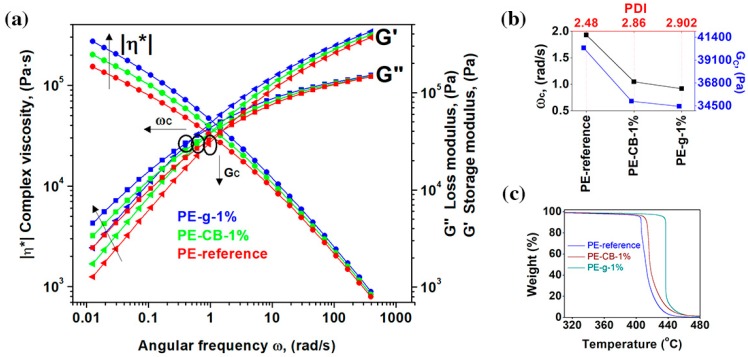
**Thermal stability and rheological behaviours of the multimodal-HDPE and its nanocomposites** (**a**) Dynamic frequency sweep measurements of the multimodal-HDPE reference (PE), PE-CB-1% and PE-g-1%, measured at 190 °C. (**b**) The polydispersity index (PDI) of the neat polymer and its nanocomposites, calculated based on the crossover frequency point (*ω*_C_) and the crossover modulus point (*G*_C_) in a log-log scale. (**c**) Thermogravimetric thermograms of the neat multimodal-HDPE, PE-CB-1% and PE-g-1%, in N_2_ atmosphere.

The dispersion and distribution of the nanofillers within the polymer matrix were further investigated by the rheological measurements [40,46,47,48]. The influence of the nanofillers on the viscoelastic response of the polymer is revealed from the change in the absolute values of the storage (*G*′) and loss (*G*′′) moduli, as well as their frequency dependence [40,46,47,48]. The pseudoplastic, non-Newtonian behaviour of the viscoelastic polymer is presented in Figure 2a. At a high shear rate, all the polymers exhibited shear thinning behaviour, which resulted in a decrease of extensional viscosity. The melt viscosity of the neat multimodal-HDPE increased with the addition of the both nanofillers, though the relative increase gradually lessened at a high shear rate.

The presence of the nanofillers considerably increased the pseudoplasticity at a low shear rate. At the angular frequency of 0.0154 rad/s, the complex viscosity of the neat multimodal-HDPE increased from 0.19 MPa·s, to 0.34 MPa·s and 0.25 MPa·s at 1 wt.% loading of graphene and carbon black, respectively. Similarly, the loss and storage moduli of the neat polymer increased by a value of ~36% and ~32% with 1 wt.% loading of carbon black and by a value of ~99% and ~78% at 1 wt.% loading of graphene, respectively. The greater amount of storage and loss moduli of the reinforced polymer is attributed to the formation of a strong interfacial bonding between the polymer matrix and the high-modulus nanofillers, which has accordingly reduced the loss tangent, therefore the polymer became more elastic [8,13,28,29,30]. The thorough dispersion and distribution of the nanofillers, as well as the strong interfacial bonding, led to a decrease in the degree of the chain mobility of the polymers, thus suppressing the shear flow of the polyethylene macromolecular chains [6]. 

As shown in Figure 2b, the crossover modulus point (*G*_C_) and crossover angular frequency point (*ω*_C_) of the reinforced polymers have shifted to a lower range. The addition of 1 wt.% of graphene decreased the *G*_C_ and *ω*_C_ of multimodal-HDPE by a value of 15% and 53%, respectively, while it decreased by a value of 13% and 46% with the 1 wt.% loading of the carbon black. These differences can be attributed to the specific surface area which each nanofiller can offer the polymer matrix through its specific dimensions [6,25]. The shift of the *ω*_C_ to the lower region indicates that the reinforced polymers exhibited higher average molar mass and/or branched (entanglement) molecules [8,40]. The later shift observed in PE-g can be attributed to formation of the jammed network structure, arising from the thorough dispersion and distribution of graphene platelets within the polymer matrix, which probably constrained the movement of the polymer chains [8,14,48,49]. Nevertheless, the shift of the *G*_C_ to the lower region upon the addition of nanofillers possibly arose also from exposing the neat polymer to a high temperature, under a combination of high shear and elongation forces, for a prolonged period of time. The weight average molar mass (Mw) and z-average molar mass (Mz) of the neat multimodal-HDPE powder decreased from 280 kg/mol and 2099 kg/mol, to 207 kg/mol and 1131 kg/mol, respectively, after being extruded in the conditions described in the experimental section (see the supplementary Table S2). Thus, the nanofiller-polymer likely induced an antioxidant synergistic effect, protecting the polymer from degradation [9,10,12,16] This is evident from the increase in the polydispersity index (PDI) of the reinforced polymers, compared with the neat multimodal-HDPE, for example, the larger is the PDI, the broader the molecular weight distribution. The advantages of synergistic effect of nanofillers are further confirmed via thermogravimetric analyses, represented in Figure 2c. 

As shown from the TGA thermograms in Figure 2c and Table 1, the onset degradation temperature of the polymer reinforced with 1 wt.% loading of graphene increased significantly, by more than 32 °C, while a temperature increase of only 6 °C was achieved with 1 wt.% loading of carbon black. The onset degradation temperature at 5% mass loss (T5%) of the neat polymer, increased from 405 °C to 410 °C with 1 wt.% loading of carbon black but to ~435 °C with 1 wt.% loading of graphene. This indicates that the nanofillers acted as a thermal barrier and improved the thermal stability of the polymers. The large aspect ratio of graphene, with platelet structure, likely offered a larger interfacial surface with the polymer matrix, which in turn slowed the diffusion of the decomposition products from a jammed network structure created in the nanocomposite [8,18,19,50,51]. Therefore, the homogenous dispersion and distribution of the graphene platelets, as well as strong interfacial bonding, are likely capable of forming a continuous network-structured protective layer, which notably reduces the heat release rate during the pyrolysis process [8,17,18,50,52]. However, the dimensional structure of the carbon black may have rendered it unable to form a continuous interconnected network structure in the polymer matrix.

**Figure 3:**
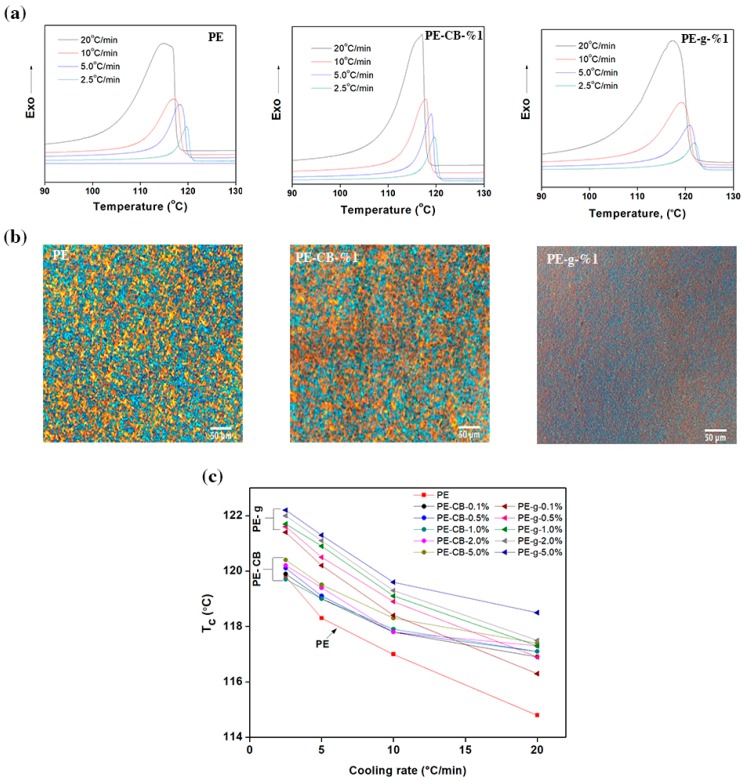
**The effect of carbon black and graphene on the crystallisation kinetics of the multimodal-HDPE.** (**a**) Non-isothermal DSC traces of the neat multimodal-HDPE, PE-CB-1% and PE-g-1%. (**b**) PLM micrographs represent the effect of graphene and carbon black on the spherulitic morphology of the multimodal-HDPE. (**c**) Effect of carbon black and graphene fillers on the peak crystallisation temperature (*T*_c_) of the multimodal-HDPE at different cooling rate.

Figure 3a and the supplementary Figure S3, show the differential scanning calorimetry (DSC) scans recorded at different cooling rates for the neat multimodal-HDPE and its nanocomposites. The exothermic crystallisation events were quenched from the molten state at constant rates of 2.5, 5, 10, 20 °C/min and the magnitude of parameters is summarised in Table 2 and the supplementary Table S1. As evident from the DSC thermograms, with an increasing cooling rate, the peak crystallisation temperature (*T*_c_) exhibited broader shape and shifted gradually to a lower temperature. The value of *T*_c_ increased from 114.8 °C to 119.8 °C for pristine multimodal-HDPE, when decreasing the cooling rate from 20 to 2.5 °C/min. In general, nucleation at a lower degree of undercooling tends to be sporadic and only a relatively small number of nuclei are obtained during the melt crystallisation [36]. At higher cooling rates, the time interval becomes sufficiently shorter in order that the random tangled molecules in the melt to align, form nuclei throughout the melt and then grow by the addition of further molecular chains [2,6,11,36,51].

A rapid decrease in temperature is accompanied by an increase in the viscosity, thereby the transport of material to the growth point becomes more difficult and eventually reduces the growth rate [6]. Hence, a higher degree of supercooling was required to initiate the crystallisation process, in such a way that the exothermic peaks became broader. In addition, the associated enthalpy of crystallisation (Δ*H*_c_) decreased where the cooling rate was elevated, as indicated in Table 2 and supplementary Table S1. The recorded enthalpy change by DSC is normally quoted as the amount of energy associated with the exotherm or endotherm per unit mass of the material analysed [6,36]. Likewise, thermal conductivity and annealing effects vary upon cooling rates [53,54,55,56,57]. The gradual decrease of the enthalpy upon the addition of nanofiller can be attributed to the proportional diminution (dilution effect) of the polyethylene concentration in the nanocomposite, as a result of the linear relationship between the latent heat and mass percentage [53,54,55,56,57]. Furthermore, the reduction of the polymer macromolecules’ freedom in the vicinity of the nanofillers, could result in less entropy and thereby decreases the enthalpy change. This can be due to either interfacial interaction or chain confinement induced by the continuous network-structure formed between the closely packed nanoparticles.

At a relatively high temperature, just below the melting temperature, the sufficient thermal energy available allows the necessary motion to take place and release the residual stresses. Therefore, the lamella crystals become thicker when held at high temperature for a longer time (annealing effects) [6,36,53,54,55,56,57]. Accordingly, the gradual decrease in Δ*H*_c_ at higher cooling rates, can also be attributed to thermal conductivity limitations, as well as lowered annealing effects. At a given cooling rate, *T*_c_ of multimodal-HDPE filled with graphene shifted to higher temperatures, as shown in Table 2 and supplementary Table S1. As shown in Figure 3b, the relative shift of *T*_c_ was clearly evident at the lowest graphene content. Afterward, the shift continued to gradually ascend as the concentration of graphene was increased, accompanied by a broadening of the peak (see Figure 3a and the supplementary Figure S3). For example, the value of *T*_c_ increased from 114.8 °C to 116.3 °C and 117.3 °C for pristine HDPE filled with 0.1 and 1.0 wt.% of graphene respectively, at the cooling rate of 20 °C/min. These changes in crystallisation behaviour can be attributed to the nucleation that took place heterogeneously on the distributed nanofillers bodies throughout the polymer matrix [6,11,36]. The foreign surfaces introduced by nanofillers reduced the barrier activation energy required to create a new surface and so lowered the degree of undercooling. This latter subject will be discussed further later in this study. On the other hand, the relative shift of *T*_c_ increased marginally at the lowest content of carbon black, except for the cooling rate of 2.5 °C/min, where no change was observed. Nonetheless, at higher concentrations the value of *T*_c_ remained somewhat unchanged.

A polarised light microscope was used to examine the spherulitic morphology of the polymers, as shown in Figure 3b. The spherulites in the polymer filled with 1 wt.% of graphene became smaller, denser and homogenously distributed throughout the polymer matrix. On the other hand, the size of spherulites after incorporating 1 wt.% of carbon black became relatively larger and distributed within the polymer matrix almost uniformly. This implies that the nanofillers were homogenously dispersed and distributed throughout the polymer matrix. The spherulite radius *r* is related to the time (*t)* and spherulite growth rate (*v)* through an equation in the form of *r* = *vt,* which is valid until the spherulites touch each other [6,36,58,59]. The decrease in spherulite sizes indicates that graphene fillers lowered the growth rate of the polymer, while the situation is apparently reversed where the carbon black is presence. A uniform distribution of the spherulite sizes can generally lead, for example, to a reduction in the post extrusion shrinkage, post-mould shrinkage and/or warpage, as well as a greater transparency by reducing the size of the scattering centres [11].

The relative degree of crystallinity (X_T_) as a function of crystallisation temperature can be calculated using the following equation

**Figure 4:**
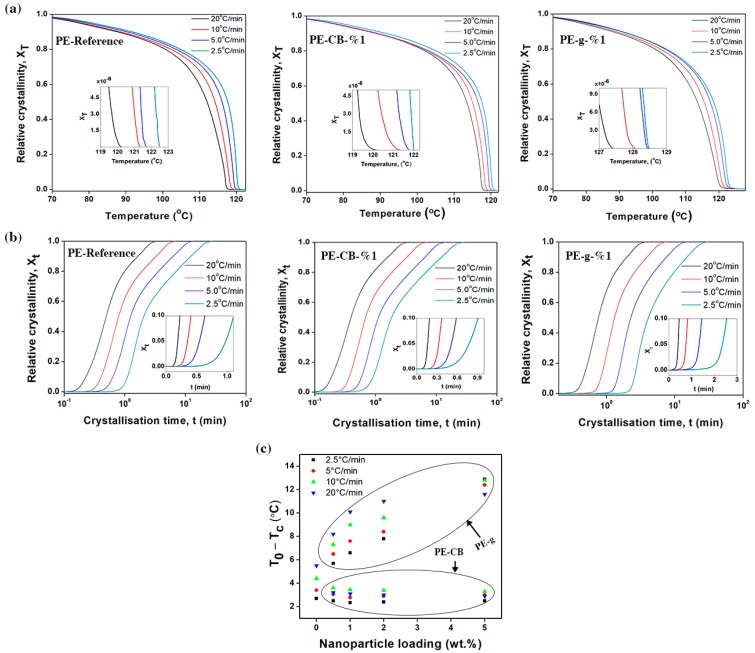
**The effect of carbon black and graphene on non-isothermal crystallisation kinetics of the multimodal-HDPE.** (**a**) Relative crystallinity evolution as a function of crystallisation temperature ($X_{T}$) for the neat multimodal-HDPE, PE-CB-1% and PE-g-1%, occurred under non-isothermal conditions. The onset temperature of crystallisation at different cooling rates are presented in the inset. (**b**) Relative crystallinity evolution as a function of crystallisation time ($X_{t}$) for the neat multimodal-HDPE, PE-CB-1% and PE-g-1%, occurred under non-isothermal conditions. The incubation period at different cooling rates are shown in the inset. (**c**) Effect of carbon black and graphene on (*T*_0_ − *T*_c_) of the multimodal-HDPE at different cooling rates.

(3) XT =∫T0T(dH)(dT)dT∫T0Tf(dH)(dT)dT

where *dH*/*dT* is the enthalpy of crystallisation evolved within an infinitesimal temperature range, *T*_0_ is the onset crystallisation temperature, *T* denotes the arbitrary crystallisation temperature and *T_f_* refers to the end temperature of crystallisation [24,34,35,36,54]. The calculated relative crystallinity values of the cooling exotherms at different cooling rates are shown in Figure 4a and the supplementary Figure S4. Evidently, the crystallisation process exhibited reversed sigmoidal kinetic curves in all samples, which is common in most semicrystalline polymers as reported elsewhere [11,36]. The sigmoidal function features an induction period followed by an accelerated growth and final prominent saturation (plateau region). The lag phase and rapid growth shown in Figure 4a and the supplementary Figure S4, are often referred to as nucleation and crystal growth processes respectively [11,28], whereas the following slow crystallisation phase is attributed to the presence of secondary crystallisation [60]. Crystallisation occurred at a lower temperature when the cooling rate was increased. At a given cooling rate, *T*_0_ of the multimodal-HDPE filled with graphene shifted significantly to higher temperatures, as shown in Table 2 and supplementary Table S1. The relative shift of *T*_0_ was clearly evident even at the lowest graphene content (0.1 wt.%) and continued to increase gradually as the concentration of graphene grew. For example, the value of *T*_0_, increased from 120.3 °C to 123.6 °C for PE-CB-1% and to 127.4 °C for PE-g-0.1%, at the cooling rate of 20 °C/min. However, the relative value of T_0_ remained almost the same for the PE-CB, regardless of the concentration of carbon black.

The difference between the onset and the peak crystallisation temperatures (*T*_0_ − *T*_c_) is widely used as an indicator of spherulitic growth rate; the larger the difference, the lower the rate of growth in the spherulites [60,61,62]. The values of (*T*_0_ − *T*_c_) are listed in Table 2 and in the supplementary Table S1. As shown in Figure 4c, addition of graphene caused the value of (*T*_0_ − *T*_c_) to increase. With a 1wt.% loading, it was increased by 144%, 121%, 105% and 84% at the cooling rate of 2.5, 5, 10, and 20 °C/min, respectively. The value of (*T*_0_ − *T*_c_) gradually became larger with increasing graphene content. On the other hand, the relative value of (*T*_0_ − *T*_c_) decreased at the lowest content of carbon black, especially at the cooling rate ≥ 10 °C/min and thereafter remained invariable at higher concentrations. These results are in concurrence with the PLM images, that is, the bigger the spherulite radius, the greater the rate of spherulitic growth (*r* = *vt).* In non-isothermal crystallisation, the arbitrary crystallisation temperature *T* is associated with the crystallisation time t through the following equation of the form

(4) $$t=\;\frac{\left|T_{o}-T\right|}{\Phi }$$

where Φ is the cooling rate [11]. The relative degree of crystallinity can accordingly be transformed as a function of time ($X_{t}$) by converting the temperature on the x-axis to time scale, as shown in Figure 4b and supplementary Figure S5.

**Figure 5:**
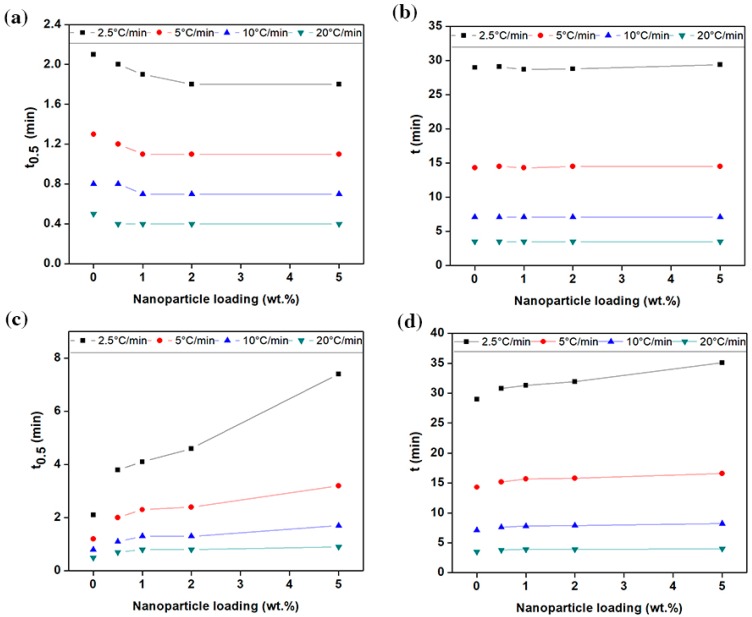
The influence of (**a**,**b**) carbon black and (**c**,**d**) graphene fillers on t_0.5_ and overall crystallisation t of the multimodal-HDPE at different cooling rates.

The magnitude of parameters obtained from Figure 4b and Figure S5 are listed in Table 2 and in the supplementary Table S1. At a given cooling rate, the time taken to complete the crystallisation *t*, relatively increased for the polymers filled with graphene, especially when decreasing the cooling rate. The value of *t* grew linearly, in concurrence with the increase in the concentration of graphene (see Figure 5c,d). With a 1 wt.% loading, for example, it was increased from 3.5, 7.1, 14.3 and 29 min to 3.9, 7.8, 15.7 and 31.3 min, at the cooling rate of 2.5, 5, 10, 20 °C/min, respectively. Interestingly, the time required to reach 50% of relative crystallinity (*t*_0.5_) extended significantly with the addition of graphene, especially at the cooling rate of ≤10 °C/min. As a further example, in PE-g-1%, the value of *t*_0.5_ increased from 0.5, 0.8, 1.2 and 2.1 to 0.8, 1.3, 2.3 and 4.1 min, at the cooling rate of 2.5, 5, 10, 20 °C/min, respectively. The marginal increase in the value of (*t* − *t*_0.5_), indicates that the time-lag occurring as a result of a longer incubation period. On the other hand, a slight decrease in *t*_0.5_ and *t* was generally observed with the addition of carbon black, as shown in Figure 5a,b. The value of *t*_0.5_ remained almost saturated when the carbon black content reached 1 wt.% and higher. These results are in agreement with the PLM images, that is, the spherulite radius is directly proportional to the rate of spherulitic growth (*r* = *vt).* This means that the crystallisation kinetics of PE-CB and PE-g were dominated by the nucleation process. As graphene has a huge surface area, it has introduced a large number of nucleation sites through which the crystallisation initiated and occurred at higher temperatures. The effect of graphene is consistent with most of the results reported in the literature on the effect of nucleating agents upon the behaviour of crystallisation in HDPE, with the exception of the declining rate of the crystallisation [11]. Nonetheless, a decrease in the crystallisation rate was observed with nanofillers that behave like graphene and produce a jammed-network structure in polymers [13,17,24,26,34,47,63]. The three-dimensional networks (entanglements) induced by the graphene platelets possibly presented an obstacle to crystallisation and led the polymer molecules to adjust their configuration over a longer period of time. This suggests why the induction time was longer, especially as the nucleation tends to be sporadic at reduced degree of undercooling [6,27]. Furthermore, the increase of the melt viscosity caused by graphene platelets, through the three-dimensional network structure and/or synergetic effect advantages, made the transport of the materials to the growth point more difficult, resulting in a decreased growth rate [59,64]. F. C. Chiu et al., found that the density of nuclei was higher for the polyethylene with higher molar mass, so this could be another reason for the increase in *T*_0_ [12,64]. Contrastingly, carbon black was shown to have little effect on the crystallisation kinetics of multimodal-HDPE, conceivably because of its surface properties.

Carbon black acting as a nucleating agent in polyethylene has never yet been reported according to the author’s knowledge. Many studies, such as those by S. Song et al. [1], A. Krumme et al. [2], T. Wu et al. [5], L. Balzano et al. [20], Y. An et al. [21], I. Dukovski and M. Muthukumar [23] and M. Gahleitner et al. [65], reported on the effect of the MWD breadth, MWD mode and the long chain molecules on the crystallisation behaviour of the polymers. Provides studies have shown that the presence of the high molar mass chains in a polyethylene with a bimodal or broad MWD can lead the crystallisation to occur at a higher temperature through an action of fluid shear. It is well-established that the specific volume of the polymer decreases upon cooling and thus a certain degree of shear flow is expected to be induced in the system. A pre-existing shear flow may also have been introduced in the compounding extruder and/or in the compression moulding during samples preparation for analysis. As a consequence, the relative change in the crystallisation kinetics of PE-CB is suggested to be mainly due to the synergistic advantage induced by carbon black. The incorporation of carbon black protected the MWD and/or the long chain molecules from becoming narrower or shorter, respectively. This is consistent with the results published by J. Wang et al. on a synergistic advantage obtained from the addition of carbon nanotubes on the crystallisation kinetics of isotactic polypropylene [15].

Using an appropriate model to account for the varying temperatures and cooling rates allows better understanding of the crystallisation kinetics under typical non-isothermal conditions. Various scientists, such as Ziabicki, K. Nakamura et al., Jeziorny, Ozawa, R. M. Patel et al., Dietz, T. Liu et al., have proposed numerous models to describe the non-isothermal crystallisation kinetics of semicrystalline polymers [11,22,23,38]. Among these models, a model proposed by T. Liu et al. was the most acclaimed and most successful approach in explaining the non-isothermal crystallisation kinetics of polyethylene and nanofillers-based polyethylene nanocomposites, such as carbon nanotubes, carbon black, graphene, diamond, graphite, CaCO_3_, SiO_2_, TiO_2_ [11,24,25,26,27,28,29,38]. The study derived a new kinetic equation by combining the Avrami equation ($X_{t}\;=\;1\;-\;\exp\left(-Z_{t}t^{n}\right)$) with the Ozawa equation $(X_{T}=(1-\exp\left[-\frac{K_{T}}{\Phi^{m}}\right]$)), where $Z_{t}$ is the overall crystallisation rate constant as a function of time, exponent *n* is a kinetic constant, $K_{T}$ is a cooling function related to the rate of overall crystallisation and changes as a function of temperature and m is the Ozawa exponent based on the dimensions of the crystal growth [38]. Rearranging and combining the Ozawa and Avrami equations leads to a kinetic equation that provides a relationship between the cooling rate Φ and arbitrary crystallisation temperature T, namely

(5) $$\log Z_{t}\;+n\;\log\;t=\log\;K_{T}-m\;l$$

and can further be rewritten as

(6) $$\log\Phi =\log F_{T}-a\log t$$

where the parameter $F_{T}$ = $\left[K_{T}/Z_{t}\right]$^1/*m*^ refers to the cooling or heating rate required to achieve a certain relative degree of crystallinity at a unit crystallisation time and a is the ratio of Avrami exponent *n* to Ozawa exponent *m*, that is, *a* = *n*/*m*.

**Figure 6:**
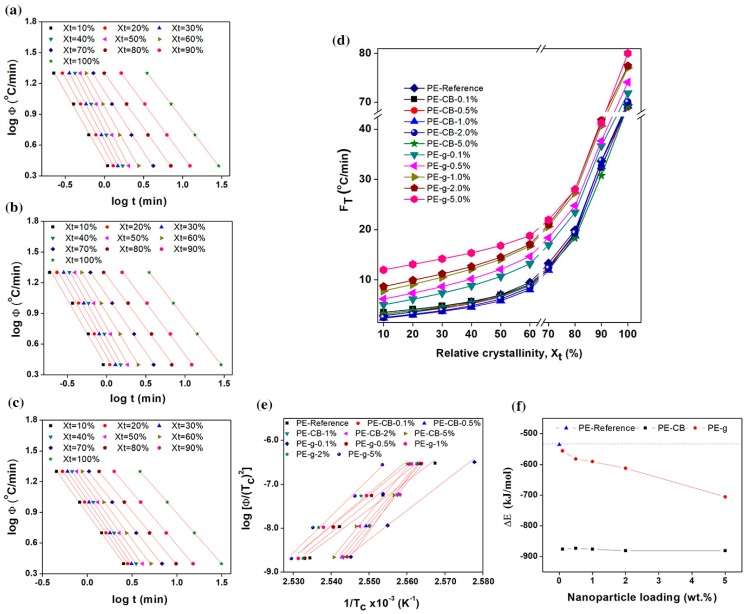
**Plots of combinedAvrami-Ozawa and H. E. Kissinger equations**. Plots of log Φ versus log *t* from the combined Avrami-Ozawa equations for (**a**) neat multimodal-HDPE, (**b**) PE-CB-1% and (**c**) PE-g-1%, during the non-isothermal crystallisation. (**d**) The kinetic parameter $F_{T}$ as a function of relative degree of crystallinity, for the multimodal-HDPE and its nanocomposites with different nanofiller loadings of 0.1, 0.5, 1, 2, 5 wt.%. Plot of (**e**) log $[\Phi /\left(T_{c}{)}^{2}\right]$ versus 1/$T_{c}$ with r^2^ > 0.985 and (**f**) Δ*E* values as a function of nanoparticle concentration obtained from the H. E. Kissinger equation for the neat multimodal-HDPE, PE-CB and PE-g.

Parameters $F_{T}$ and −*a* can be determined from the intercept and slope of the log φ versus log *t* plot respectively, as shown in Figure 6a–c and supplementary Figure S6. According to Equation (6), the series of linear relationships with coefficient of determination r^2^ ≥ 0.998, was observed at a given relative degree of crystallinity, indicating that the T. Liu et al. model could aptly describe the non-isothermal crystallisation kinetics of the multimodal-HDPE, PE-g and PE-CB. The kinetic parameter *F_T_* as a function of relative degree of crystallinity and the nanofiller content, is shown in Figure 6d, for the multimodal-HDPE, PE-CB and PE-g, representing the cooling rate required for every sample to achieve a certain degree of relative crystallinity at a unit crystallisation time. It was directly proportional to the relative degree of crystallinity, while values of *a* were almost the same. The marginal changes in the values of *a*, ranged from 1 to 1.3, which also indicates that the combined Avrami-Ozawa equations suitably describes the non-isothermal crystallisation kinetics of the multimodal-HDPE, PE-g and PE-CB, as reported elsewhere [11,24,25,26,27,28,29,30,33,34,35,36]. As shown and discussed earlier in the present study, $X_{t}$ became larger, at a unit crystallisation time, by increasing the cooling rate and the crystallisation roll-off occurred at around a 70% degree of crystallinity, which is characteristic of slow or secondary crystallisation. In general, *F_T_* values of the neat HDPE and PE-CB were almost the same and increased in a similar pattern across the entire degree of relative crystallinity. A slight decrease in *F_T_* was observed when the polyethylene was loaded with ≥1 wt.% carbon black content, which is consistent with the results observed earlier in this study.

The *F_T_* parameter of PE-CB-1% decreased from 2.8, 7 and 69.5 °C/min to 2.4, 5.8 and 69.2 °C/min at *X*_t_ of 10%, 50% and 100%, respectively. This is a confirmation that carbon black did not form a continuous network structure, most probably because of its dimensionality. However, the relative increase in *F*_T_ was clearly evident even at the lowest graphene content and continued to gradually rise as the concentration of graphene became larger, especially at *X*_t_ < 70%. The *F*_T_ value of the multimodal-HDPE filled with 1 wt.% graphene, increased from 2.8, 7 and 69.5 °C/min to 7.8, 14.1 and 77.1 °C/min at *X*_t_ of 10%, 50% and 100%, respectively. This is indicative of a slower crystallisation process compared with the neat HDPE and/or HDPE filled with CB and it is related to the difficulty of crystallisation process to progress further due to the entangled-network structure induced by graphene platelets. These results are consistent with what was observed and discussed earlier in this study. Accordingly, knowledge of the conditions affecting the crystallisation kinetics is crucial for optimising the processing parameters, as this can affect the final properties of the product which includes warpage/shrinkage control, mechanical and optical clarity or an alteration of the cycle time in moulding or extrusion [11]. In pipe manufacturing, for example, the thermal gradient across the pipe wall generates residual stress within the pipe, as the crystallisation in the core region is slower than the inner and outer surfaces that are cooled down by a water spray [1,3]. A replacement of the nanofiller, such as carbon black by graphene, requires a consequent optimisation of the processing parameters.

At a given cooling rate, the relative degree of crystallinity at the peak crystallisation temperature ranges between 11% and 30% for all the polymers. The mean activation energy barrier (Δ*E*) required for the molecular chains to transport from the melt state to the growing crystal surface, in non-isothermal systems, was calculated in this study, using the following H. E. Kissinger equation [19,34,35,36,38,39]

(7) $$\frac{d\left[\log\frac{\Phi }{T_{c}^{2}}\right]}{d\left(\frac{1}{T_{c}}\right)}=-\frac{\Delta E}{R}$$

where *R* is the universal gas constant. The higher the value of Δ*E*, the more difficult the transportation of macromolecular chains to the growing surface. These can generally result either from higher viscosity or restriction in the polymer chains as previously discussed in the present study. The activation energies obtained from the slope of the straight-lines in a $\log(\frac{\Phi }{T_{c}^{2}})$ versus $\frac{1}{T_{c}}$ plot in Figure 6e, are plotted as a function of nanoparticle content in Figure 6f. The dashed line represents the Δ*E* of the neat multimodal-HDPE, which was −535 kJ/mol. The value of Δ*E* decreased gradually to −556, −582, −591, −612 and −705 kJ/mol when increasing the concentration of graphene to 0.1, 0.5, 1, 2, 5 wt.%, respectively. However, the relative value of Δ*E* decreased significantly to −876 kJ/mol at the lowest carbon black content of 0.1 wt.% and remained almost saturated at higher concentration. In general, the decrease in the activation energy barrier is attributable to the role of the nanoparticles as a nucleating agent. The results indicate that graphene is likely to perform two functions in the multimodal-HDPE matrix. The platelets acted as nucleating agents and accelerated the primary nucleation, which was manifested by the gradual decrease in Δ*E* and the increase in *T*_0_ and *T*_c_. However, the presence of the jammed network structure slowed the diffusion of the polymer chains into the crystalline lattice, thus lowering the overall crystallisation rate, as confirmed by the increase in *F*_T_, *t*_0.5_ and *t*. On the other hand, the change in crystallisation kinetics of the multimodal-HDPE, associated with the addition of carbon black, is suggested to be a result of the synergistic effects. This occurs where the longer macromolecular chains and broader MWD accelerated the crystallisation of PE-CB as confirmed from the values of Δ*E*, *t*_0.5_, *T*_c_, *F*_T_, as well as rheological results.

## 4. Conclusions

The crystallisation kinetics of the multimodal-HDPE behaved differently with the addition of 0.1 to 5 wt.% carbon black or graphene, under non-isothermal conditions. The non-isothermal crystallisation behaviour of the PE-g nanocomposites relied heavily on both the graphene content and cooling rate. The relative peak crystallisation temperature of PE-g increased with decreasing the cooling rate for a given graphene content and increased gradually with an increase in the concentration of graphene at a given cooling rate. Incorporation of graphene caused a change in the mechanism of nucleation and crystal growth of multimodal-HDPE crystallites, with the effect being more evident at the lowest graphene content. At a given cooling rate, the nucleation initiated at a higher temperature and continued to increase with the graphene content. Similarly, the incubation period as well as the time required to reach 50% of relative crystallinity *t*_0.5_ increased significantly with the addition of graphene and continued to increase gradually with an increasing content of graphene. However, the spherulitic growth rate decreased at the lowest graphene content and continued its gradual descent with the increasing amount of graphene content. On the other hand, the non-isothermal crystallisation behaviour of the PE-CB nanocomposites relied on the cooling rate only and the effect of carbon black content as a nucleating agent was found to be marginal, with the exception of the peak crystallisation temperature which increased gradually in concurrence with the carbon black content. At a given cooling rate, the relative onset crystallisation temperature of PE-CB remained almost unchanged with the addition of the carbon black. Also, a slight increase in the spherulitic growth rate accompanied a relative decrease in t_0.5_ which was observed upon addition of 0.1% carbon black and these remained almost unchanged with further addition.

Combined Avrami-Ozawa equations proposed by Liu et al. was found to be effective in describing the non-isothermal crystallisation kinetics of the multimodal-HDPE, PE-CB and PE-g nanocomposites. The cooling rate required to achieve certain degree of crystallinity increased gradually as the graphene content increased. The relative kinetic parameter *F*_T_ of the multimodal-HDPE filled with 1 wt.% graphene increased from 2.8, 7.0 and 69.5 °C/min to 7.8, 14.1 and 77.1 °C/min at *X*_t_ of 10%, 50% and 100%, respectively. However, a slight decrease in *F*_T_ values was observed when the multimodal-HDPE loaded with ≥1 wt.% carbon black content. Using the H. E. Kissinger equation, the activation barrier energy of the multimodal-HDPE decreased gradually with an increasing the graphene concentration, while it rose by 64% at the lowest carbon black content. However, the value of Δ*E* remained almost unchanged as the content of carbon black increased. For PE-g, graphene platelets acted as nucleating agents and introduced foreign surfaces, which in turn reduced the barrier activation energy required for the crystallisation. However, the presence of the jammed network structure introduced by graphene platelets retarded the diffusion of the polymer chains into the crystalline lattice and thus slowing the primary crystallisation. Contrastingly, carbon black was shown to have little effect as a heterogeneous nucleating agent on the crystallisation kinetics of multimodal-polyethylene, possibly because of its surface properties.

## Figures and Tables

**Table 1:** TGA data analysis of the neat multimodal-HDPE, PE-CB-1% and PE-g-1%.

Sample	T5%, (°C)	T30%, (°C)	T50%, (°C)	T80%, (°C)
PE-reference	405	406.8	409	419.3
PE-g-1%	435	437	437	438
PE-CB-1%	409.8	415	417	429

T5%, T30%, T50% and T80%, are the onset temperatures at 5%, 30%, 50% and 80% mass loss, respectively.

**Table 2:** Nano-isothermal crystallisation parameters of multimodal-HDPE, PE-g and PE-CB.

Sample	Φ, (°C/min)	*T*_0_, (°C)	*T*_c_, (°C)	*T*_0_−*T*_c_, (°C)	*T*_0.5_, (°C)	*t*_0.5_, (min)	Δ*H*_c_, (J/g)	*T*_f_, (°C)	*t*, (min)	*X*_c_ (%)
Neat HDPE	20	120.3	114.8	5.5	112.0	0.5	184	50.1	3.5	70
	10	121.4	117.0	4.4	114.2	0.8	187	50.2	7.1	71
	5	121.7	118.3	3.4	116.1	1.3	193	50.3	14.3	71
	2.5	122.5	119.8	2.7	117.7	2.1	199	50.1	29.0	71
PE-CB-1.0%	20	120.2	117.1	3.1	113.5	0.4	179	50.3	3.5	69
	10	121.4	117.9	3.5	115.1	0.7	184	50.1	7.1	70
	5	121.8	119.0	2.8	116.6	1.1	188	50.1	14.3	70
	2.5	122	120.1	2.3	117.6	1.9	191	50.2	28.7	70
PE-g-1.0%	20	127.4	117.3	10.1	113.5	0.8	176	49.9	3.9	69
	10	128.1	119.1	9.0	115.5	1.3	181	50.2	7.8	69
	5	128.4	120.9	7.5	117.4	2.3	186	50.1	15.7	69
	2.5	128.5	121.7	6.6	118.4	4.1	191	50.2	31.3	70

Φ is the cooling rate; *T*_0_, *T*_c_ and *T*_f_ denote for the onset, crystallisation, end crystallisation temperatures, respectively; *T*_0.5_ and *t*_0.5_ are the temperature and time required to reach 50% of relative crystallinity, respectively.

## Supplementary Materials

The following are available online at http://www.mdpi.com/2079-4991/9/1/110/s1, Figure S1: Light microscopy images of the multimodal-HDPE loaded with 1.0 wt.% of carbon black (left) and graphene (right). The images were processed using ImageJ software, Figure S2: TEM images of (a) carbon black and (b) graphene platelets. The images of carbon black were taken under Hitachi HT7700, at an accelerating voltage of 120 kV. Whereas JEOL JEM2100FCs Field Emission TEM, with CEOS aberration corrected illumination system at an accelerating voltage of 200 kV, was used to image the graphene platelets, Figure S3: Non-isothermal DSC traces of PE-CB and PE-g as a function of nanofiller content, Figure S4: Relative crystallinity evolution as a function of crystallisation time ($X_{T}$) for the PE-CB and PE-g at 0.1, 0.5, 2, 5 wt.%, occurred under non-isothermal conditions. The onset temperature of crystallisation at different cooling rates are presented in the inset, Figure S5: Relative crystallinity evolution as a function of crystallisation time ($X_{t}$) for the PE-CB and PE-g at 0.1, 0.5, 2, 5 wt.%, occurred under non-isothermal conditions. The incubation period at different cooling rates are presented in the inset, Figure S6: Plots of log Φ versus log *t* from the combined Avrami-Ozawa equations for the multimodal-HDPE filled with graphene or carbon black at 0.1, 0.5, 2, 5 wt.%, during the non-isothermal crystallisation, Table S1: Nano-isothermal crystallisation parameters of neat HDPE, PE-g and PE-CB, Table S2: Average molecular weights for the neat multimodal-HDPE before and after extrusion.

## References

[1] Song S., Wu P., Ye M., Feng J., Yang Y. (2008). Effect of small amount of ultra high molecular weight component on the crystallization behaviors of bimodal high density polyethylene. Polymer.

[2] Krumme A., Lehtinen A., Viikna A. (2004). Crystallisation behaviour of high density polyethylene blends with bimodal molar mass distribution: 2. Non-isothermal crystallization. Eur. Polym. J..

[3] DesLauriers P.J., McDaniel M.P., Rohlfing D.C., Krishnaswamy R.K., Secora S.J., Benham E.A., Maeger P.L., Wolfe A.R., Sukhadia A.M., Beaulieu B.B. (2005). A comparative study of multimodal vs. bimodal polyethylene pipe resins for PE-100 applications. Polym. Eng. Sci..

[4] Chatzidoukas C., Kanellopoulos V., Kiparissides C. (2007). On the production of polyolefins with bimodal molecular weight and copolymer composition distributions in catalytic gas-phase fluidized-bed reactors. Macromol. Theor. Simul..

[5] Wu T., Yu L., Cao Y., Yang F., Xiang M. (2013). Effect of molecular weight distribution on rheological, crystallization and mechanical properties of polyethylene-100 pipe resins. J. Polym. Res..

[6] Young R.J., Lovell P.A. (2011). Introduction to Polymers.

[7] Kuilla T., Bhadra S., Yao D., Hoon N., Bose S., Hee J. (2010). Recent advances in graphene based polymer composites. Prog. Polym. Sci..

[8] Ahmad I., Koziol K., Deveci S., Kim H.-K., Kumar R. (2018). Advancing the use of high-performance graphene-based multimodal polymer nanocomposite at scale. Nanomaterials.

[9] Hawkins W.L., Hansen R.H., Matreyek W., Winslow F.H. (1959). The effect of carbon black on thermal antioxidants for polyethylene. J. Appl. Polym. Sci..

[10] Mwila J., Miraftab M., Horrocks A.R. (1994). Effect of carbon black on the oxidation of polyolefins—An overview. Polym. Degrad. Stab..

[11] Seven K.M., Cogen J.M., Gilchrist J.F. (2016). Nucleating agents for high-density polyethylene—A review. Polym. Eng. Sci..

[12] (2017). The Global Market for Carbon Black Report.

[13] Kim J., Kwak S., Hong S.M., Lee J.R., Takahara A., Seo Y. (2010). Nonisothermal crystallization behaviors of nanocomposites prepared by in situ polymerization of high-density polyethylene on multiwalled carbon nanotubes. Macromolecules.

[14] Mcnally T., Pötschke P., Halley P., Murphy M., Martin D., Bell S.E.J., Brennan G.P., Bein D., Lemoine P., Quinn J.P. (2005). Polyethylene multiwalled carbon nanotube composites. Polymer.

[15] Wang J., Yang J., Deng L., Fang H., Zhang Y., Wang Z. (2015). More dominant shear flow effect assisted by added carbon nanotubes on crystallization kinetics of isotactic polypropylene in nanocomposites. ACS Appl. Mater. Interfaces.

[16] Qiu Y., Wang Z., Owens A.C.E., Kulaots I., Chen Y., Kane A.B., Hurt R.H. (2014). Antioxidant chemistry of graphene-based materials and its role in oxidation protection technology. Nanoscale.

[17] El Achaby M., Qaiss A. (2013). Processing and properties of polyethylene reinforced by graphene nanosheets and carbon nanotubes. Mater. Des..

[18] Kashiwagi T., Du F., Douglas J.F., Winey K.I., Harris R.H., Shields J.R. (2005). Nanoparticle networks reduce the flammability of polymer nanocomposites. Nat. Mater..

[19] Shan H., Lickfield G.C. (2007). Crystallization kinetics study of polyethylene. Int. J. Polym. Anal. Charact..

[20] Balzano L., Rastogi S., Peters G. (2011). Self-nucleation of polymers with flow: The case of bimodal polyethylene. Macromolecules.

[21] An Y., Holt J.J., Mitchell G.R., Vaughan A.S. (2006). Influence of molecular composition on the development of microstructure from sheared polyethylene melts: Molecular and lamellar templating. Polymer.

[22] Balzano L., Kukalyekar N., Rastogi S., Peters G.W.M., Chadwick J.C. (2008). Crystallization and dissolution of flow-induced precursors. Phys. Rev. Lett..

[23] Dukovski I., Muthukumar M. (2003). Langevin dynamics simulations of early stage shish-kebab crystallization of polymers in extensional flow. J. Chem. Phys..

[24] Shehzad F., Thomas S.P., Al-Harthi M.A. (2014). Non-isothermal crystallization kinetics of high density polyethylene/graphene nanocomposites prepared by in-situ polymerization. Thermochim. Acta.

[25] Shi X., Wang J., Jiang B., Yang Y. (2013). Influence of nanofiller dimensionality on the crystallization behavior of HDPE/carbon nanocomposites. J. Appl. Polym. Sci..

[26] Huang J.-W., Wen Y.-L., Kang C.-C., Tseng W.-J., Yeh M.-Y. (2008). Nonisothermal crystallization of high density polyethylene and nanoscale calcium carbonate composites. Polym. Eng. Sci..

[27] Mercier J.P. (1990). Nucleation in polymer crystallization: A physical or a chemical mechanism?. Polym. Eng. Sci..

[28] Adhikari A.R., Lozano K., Chipara M. (2012). Non-isothermal crystallization kinetics of polyethylene/carbon nanofiber composites. J. Compos. Mater..

[29] Jiasheng Q., Pingsheng H. (2003). Non-isothermal crystallization of HDPE/nano-SiO_2_ composite. J. Mater. Sci..

[30] Yang B., Deng Y.-L., Li G.-J., Miao J.-B., Xia R., Qian J.-S., Chen P., Liu J.-W. (2015). Solidification behavior of high-density polyethylene (HDPE) during injection molding: Correlation between crystallization kinetics and thermal gradient field. IOP Conf. Ser. Mater. Sci. Eng..

[31] Nezhad H.Y., Thakur V.K. (2018). Effect of morphological changes due to increasing carbon nanoparticles content on the quasi-static mechanical response of epoxy resin. Polymers.

[32] Miculescu M., Thakur V.K., Miculescu F., Voicu S.I. (2016). Graphene-based polymer nanocomposite membranes: A review. Polym. Adv. Technol..

[33] Muhulet A., Miculescu F., Voicu S.I., Schütt F., Thakur V.K., Mishra Y.K. (2018). Fundamentals and scopes of doped carbon nanotubes towards energy and biosensing applications. Mater. Today Energy.

[34] Banks W., Gordon M., Roe R.-J., Sharples A. (1963). The crystallization of polyethylene I. Polymer.

[35] Van Drongelen M., Roozemond P.C., Troisi E.M., Doufas A.K., Peters G.W.M. (2015). Characterization of the primary and secondary crystallization kinetics of a linear low-density polyethylene in quiescent- and flow-conditions. Polymer.

[36] Mandelkern L. (2004). Crystallisation of Polymers: Volume 2. Kinetics and Mechanisms.

[37] Hiesgen G., Saul K., Rauwendaal C. (2016). Rauwendaal. Temperature induced dimensional variation in extrusion. AIP Conf. Proc..

[38] Liu T., Mo Z., Wang S., Zhang H. (1997). Nonisothermal melt and cold crystallization kinetics of poly(aryl ether ether ketone ketone). Polym. Eng. Sci..

[39] Kissinger H.E. (1956). Variation of peak temperature with heating rate in differential thermal analysis. J. Res. Natl. Bur. Stand..

[40] Mezger T.G. (2014). The Rheology Handbook.

[41] Cebe P., Thomas D., Merfeld J., Partlow B.P., Kaplan D.L., Alamo R.G., Wurm A., Zhuravlev E., Schick C. (2017). Heat of fusion of polymer crystals by fast scanning calorimetry. Polymer.

[42] Nair R.R., Blake P., Grigorenko A.N., Novoselov K.S., Booth T.J., Stauber T., Peres N.M.R., Geim A.K. (2008). Fine structure constant defines visual transparency of graphene. Science.

[43] Cote L.J., Kim J., Zhang Z., Sun C., Huang J. (2010). Tunable assembly of graphene oxide surfactant sheets: Wrinkles, overlaps and impacts on thin film properties. Soft Matter.

[44] Albers P., Maier M., Reisinger M., Hannebauer B., Weinand R. (2015). Physical boundaries within aggregates—Differences between amorphous, para-crystalline, and crystalline Structures. Cryst. Res. Technol..

[45] Levy L., Chaudhuri I.S., Krueger N., McCunney R.J. (2012). Does carbon black disaggregate in lung fluid? A critical assessment. Chem. Res. Toxicol..

[46] Tang D., Li S., Yang J., Su J., Yang Q., Kong M., Huang Y., Liao X. (2017). Nonisothermal and isothermal crystallization behavior of isotactic polypropylene/chemically reduced graphene nanocomposites. Polym. Compos..

[47] Wu D., Cheng Y., Feng S., Yao Z., Zhang M. (2013). Crystallization behavior of polylactide/graphene composites. Ind. Eng. Chem. Res..

[48] Zhang Q., Rastogi S., Chen D., Lippits D., Lemstra P.J. (2006). Low percolation threshold in single-walled carbon nanotube/high density polyethylene composites prepared by melt processing technique. Carbon.

[49] Liu K., Andablo-Reyes E., Patil N., Merino D.H., Ronca S., Rastogi S. (2016). Influence of reduced graphene oxide on the rheological response and chain orientation on shear deformation of high density polyethylene. Polymer.

[50] Kashiwagi T. (1994). Polymer combustion and flammability—Role of the condensed phase. Symp. Combust..

[51] Xiong H., Gao Y., Li H.M. (2007). Non-isothermal crystallization kinetics of syndiotactic polystyrene–polystyrene functionalized SWNTs nanocomposites. eXPRESS Polym. Lett..

[52] Gorrasi G., Bugatti V., Milone C., Mastronardo E., Piperopoulos E., Iemmo L., Di Bartolomeo A. (2018). Effect of temperature and morphology on the electrical properties of PET/conductive nanofillers composites. Compos. Part B Eng..

[53] Valentini L., Biagiotti J., López-Manchado M.A., Santucci S., Kenny J.M. (2004). Effects of carbon nanotubes on the crystallization behavior of polypropylene. Polym. Eng. Sci..

[54] Wang W., Yang X., Fang Y., Ding J., Yan J. (2009). Preparation and thermal properties of polyethylene glycol/expanded graphite blends for energy storage. Appl. Energy.

[55] Dietz W. (2016). Effect of cooling on crystallization and microstructure of polypropylene. Polym. Eng. Sci..

[56] Fischer E.W. (1972). Effect of annealing and temperature on the morphological structure of polymers. Pure Appl. Chem..

[57] Wang W., Zhao G., Wu X., Zhai Z. (2015). The effect of high temperature annealing process on crystallization process of polypropylene, mechanical properties, and surface quality of plastic parts. J. Appl. Polym. Sci..

[58] Raimo M. (2011). Estimation of polymer nucleation and growth rates by overall DSC crystallization rates. Polym. J..

[59] Magill J.H., Li H.M. (1978). Crystallization kinetics and morphology of polymer blends of poly(tetramethyl-p-silphenylene siloxane) fractions. Polymer.

[60] Jain S., Goossens H., van Duin M., Lemstra P. (2005). Effect of in situ prepared silica nano-particles on non-isothermal crystallization of polypropylene. Polymer.

[61] Beck H., Ledbetter H. (1965). DTA study of heterogeneous nucleation of crystallization in polypropylene. J. Appl. Polym. Sci..

[62] Mareri P., Bastide S., Binda N., Crespy A. (1998). Mechanical behaviour of polypropylene composites containing fine mineral filler: Effect of filler surface treatment. Compos. Sci. Technol..

[63] Zheng W., Lu X., Wong S.-C. (2004). Electrical and mechanical properties of expanded graphite-reinforced high-density polyethylene. J. Appl. Polym. Sci..

[64] Chiu F.-C., Fu Q., Hsieh E.T. (1999). Molecular weight dependence of melt crystallization behavior and crystal morphology of low molecular weight linear polyethylene fractions. J. Polym. Res..

[65] Gahleitner M., Bernreitner K., Neil W. (1995). Influence of molecular structure on crystallization behaviour and mechanical properties of polypropylene. Polym. Test..

