# Correction: Ahmad, I.A., et al. Non-Isothermal Crystallisation Kinetics of Carbon Black-Graphene-Based Multimodal-Polyethylene Nanocomposites. *Nanomaterials*, 2019, *9*, 100

**DOI:** 10.3390/nano9030392

**Published:** 2019-03-07

**Authors:** Ibrahim A. Ahmad, Hyun-Kyung Kim, Suleyman Deveci, R. Vasant Kumar

**Affiliations:** 1 Department of Materials Science and Metallurgy, University of Cambridge, 27 Charles Babbage Rd, Cambridge CB3 0FS, UK; iaiaa2@cam.ac.uk; 2 Gwangju Bio/Energy R&D Center, Korea Institute of Energy Research (KIER), 270-25 Samso-ro, Buk-gu, Gwangju 61003, Korea; 3 Innovation Centre, Borouge Pte Ltd., PO BOX 6951, Abu Dhabi, UAE; suleyman.deveci@borouge.com

In the published paper [1], there was a typo error mistake in Equation (5), which was supposed to be expressed as “$\log Z_{t}+n\log t=\log K_{T}-m\log\Phi$” instead of “log *Zt* + *n* log *t* = log *K*_T_ − *m*l”.

The changes do not affect the scientific results. We apologize for any inconvenience caused to the readers by the error. The manuscript will be updated, and the original will remain online on the webpage for the article, including a reference to this Correction.
