# Supplementary material for: Non-Isothermal Crystallisation Kinetics of Carbon Black- Graphene-Based Multimodal-Polyethylene Nanocomposites
Source: Nanomaterials (Basel). 2019 Jan 18;9(1):110. doi: 10.3390/nano9010110 (PMC6359078; doi:10.3390/nano9010110)
Supplement: Supplementary file 1 [file nanomaterials-09-00110-s001.pdf]

*Article*

# Non-Isothermal Crystallisation Kinetics of Carbon Black- Graphene-Based Multimodal-Polyethylene Nanocomposites

**Ibrahim A. Ahmad [1], Hyun-Kyung Kim [1,2,*], Suleyman Deveci [3] and R. Vasant Kumar [1,*]**

[1] Department of Materials Science and Metallurgy, University of Cambridge, 27 Charles Babbage Rd, Cambridge CB3 0FS, UK; iaiaa2@cam.ac.uk
[2] Gwangju Bio/Energy R&D Center, Korea Institute of Energy Research (KIER), 270-25 Samso-ro, Buk-gu, Gwangju 61003, Korea
[3] Innovation Centre, Borouge Pte Ltd., PO BOX 6951, Abu Dhabi, UAE; suleyman.deveci@borouge.com
[*] Correspondence: hkk28@cam.ac.uk (H.-K.K.); rvk10@cam.ac.uk (R.V.K.);
Tel.: +44-(0)-1223-331953 (H.-K.K.); Tel: +44-(0)-1223-334327 (R.V.K.)

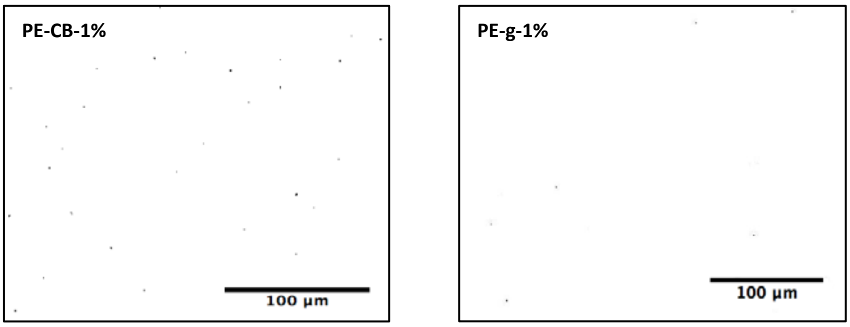


**Figure S1.** Light microscopy images of the multimodal-HDPE loaded with 1.0 wt.% of carbon black (**left**) and graphene (**right**). The images were processed using ImageJ software.

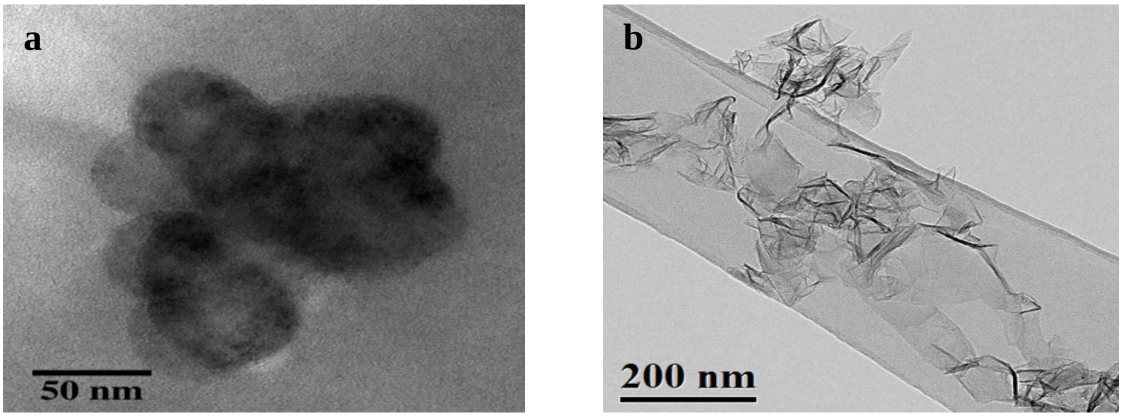


**Figure S2.** TEM images of (**a**) carbon black and (**b**) graphene platelets. The images of carbon black were taken under Hitachi HT7700, at an accelerating voltage of 120 kV. Whereas JEOL JEM2100FCs Field Emission TEM, with CEOS aberration corrected illumination system at an accelerating voltage of 200 kV, was used to image the graphene platelets.

Figure S2a shows the carbon black structure within the polymer matrix. The primary particles of carbon black, with an average particle size of ≤ 20 nm, were welded and are neither discrete nor having physical boundaries. Graphene platelets images were taken at an accelerating voltage of 200 kV using JEOL JEM2100FCs Field Emission TEM, equipped with CEOS aberration corrected illumination system, on a standard 3 mm lacey-carbon support copper grid. It was difficult to distinguish between the carbon black aggregates through the use of the latter technique, especially since they were in the powder form. Of the observed less-disturbed platelets, the average lateral dimensions were generally between 150–500 nm. The ultrathin platelets showed a predominance of edge-wrapping, wrinkling and overlapping.

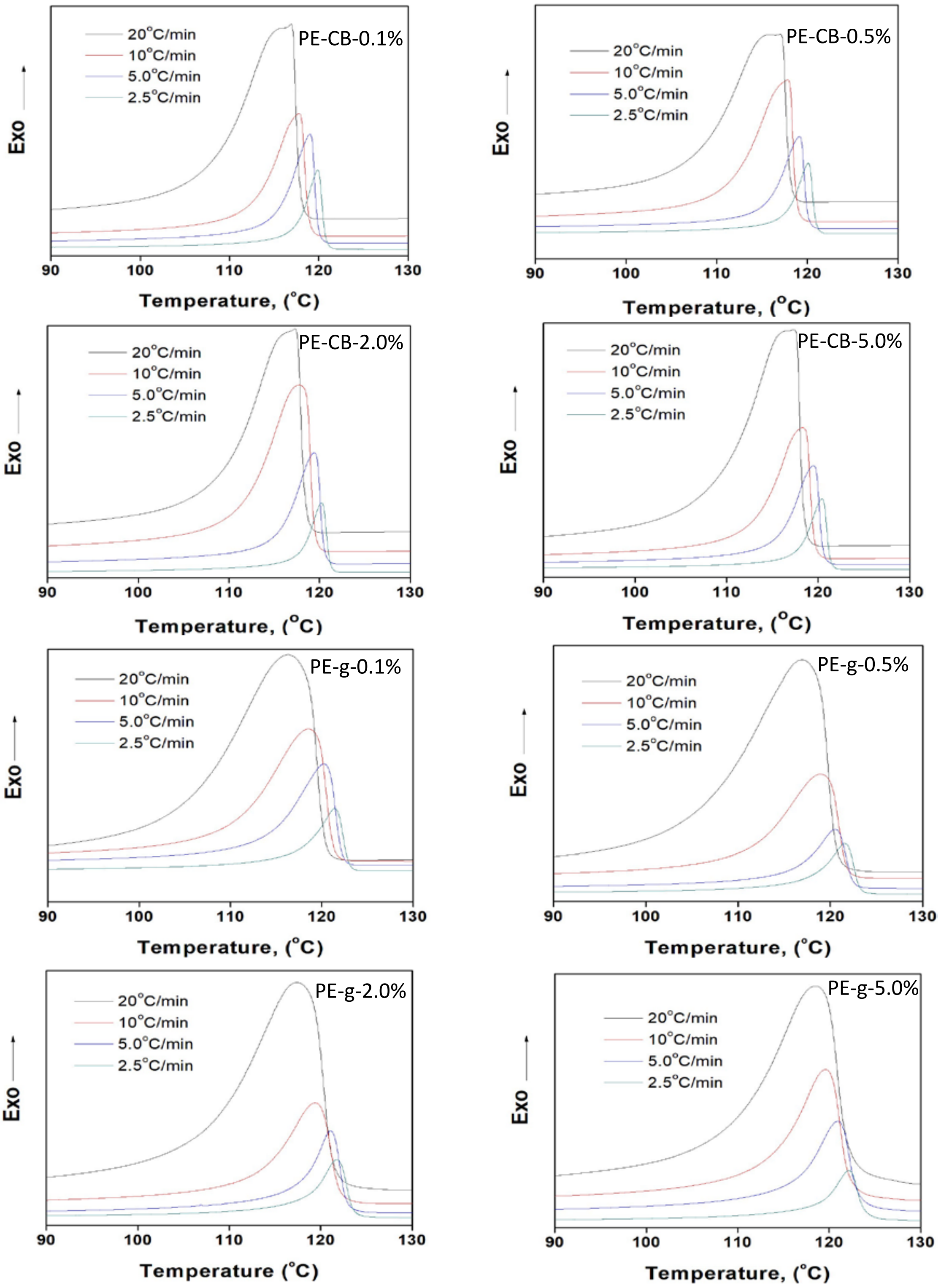


**Figure S3.** Non-isothermal DSC traces of PE-CB and PE-g as a function of nanofiller content.

**Table S1.** Nano-isothermal crystallisation parameters of neat HDPE, PE-g and PE-CB.

| Sample | Φ, (°C /min) | $T_0$, (°C) | $T_c$, (°C) | $T_0$-$T_c$, (°C) | $T_{0.5}$, (°C) | $t_{0.5}$, (min) | $\Delta H_c$, (J/g) | $T_f$, (°C) | t, (min) | $X_c$, (%) |
|---|---|---|---|---|---|---|---|---|---|---|
| **PE** | **20** | 120.3 | 114.8 | 5.5 | 112.0 | 0.5 | 184 | 50.1 | 3.5 | 70 |
| | **10** | 121.4 | 117.0 | 4.4 | 114.2 | 0.8 | 187 | 50.2 | 7.1 | 71 |
| | **5** | 121.7 | 118.3 | 3.4 | 116.1 | 1.3 | 193 | 50.3 | 14.3 | 71 |
| | **2.5** | 122.5 | 119.8 | 2.7 | 117.7 | 2.1 | 199 | 50.1 | 29.0 | 71 |
| **PE-CB-0.1%** | **20** | 120.3 | 116.9 | 3.4 | 112.8 | 0.4 | 182 | 50.0 | 3.5 | 70 |
| | **10** | 121.4 | 117.8 | 3.6 | 114.9 | 0.7 | 186 | 50.3 | 7.1 | 70 |
| | **5** | 122.5 | 119.0 | 3.5 | 116.6 | 1.3 | 190 | 50.2 | 14.5 | 70 |
| | **2.5** | 122.7 | 119.9 | 2.8 | 117.5 | 2.3 | 195 | 50.1 | 29.2 | 71 |
| **PE-CB-0.5%** | **20** | 120.3 | 117.1 | 3.2 | 112.9 | 0.4 | 181 | 50.3 | 3.5 | 69 |
| | **10** | 121.4 | 117.8 | 3.6 | 114.6 | 0.8 | 183 | 50.2 | 7.1 | 70 |
| | **5** | 122.2 | 119.1 | 3.1 | 116.7 | 1.2 | 189 | 50.2 | 14.5 | 70 |
| | **2.5** | 122.6 | 120 | 2.5 | 118.2 | 2.0 | 192 | 50.1 | 29.1 | 71 |
| **PE-CB-2.0%** | **20** | 120.3 | 117.3 | 3.0 | 113.4 | 0.4 | 178 | 50.1 | 3.5 | 69 |
| | **10** | 121.2 | 118.0 | 3.4 | 114.9 | 0.7 | 182 | 50.3 | 7.1 | 70 |
| | **5** | 122.3 | 119.4 | 2.9 | 116.9 | 1.1 | 184 | 50.1 | 14.5 | 70 |
| | **2.5** | 122.6 | 120.2 | 2.4 | 118.3 | 1.8 | 188 | 50.6 | 28.8 | 70 |
| **PE-CB-5.0%** | **20** | 120.3 | 117.4 | 2.9 | 113.7 | 0.4 | 172 | 50.1 | 3.5 | 67 |
| | **10** | 121.6 | 118.4 | 3.3 | 115.6 | 0.7 | 174 | 50.3 | 7.1 | 67 |
| | **5** | 122.5 | 119.5 | 3.0 | 117.1 | 1.1 | 176 | 50.2 | 14.5 | 68 |
| | **2.5** | 122.9 | 120.4 | 2.5 | 118.2 | 1.8 | 177 | 50.1 | 29.4 | 69 |
| **PE-g-0.1%** | **20** | 123.6 | 116.3 | 7.3 | 112.6 | 0.6 | 183 | 50.1 | 3.7 | 69 |
| | **10** | 124.4 | 118.4 | 5.9 | 114.8 | 1.0 | 187 | 50.2 | 7.4 | 70 |
| | **5** | 125.2 | 120.2 | 5.0 | 116.5 | 1.8 | 190 | 50.3 | 15.0 | 70 |
| | **2.5** | 126.6 | 121.4 | 5.2 | 118.0 | 3.5 | 198 | 50.2 | 30.5 | 70 |
| **PE-g-0.5%** | **20** | 125.1 | 116.9 | 8.2 | 112.9 | 0.7 | 179 | 50.1 | 3.8 | 69 |
| | **10** | 126.2 | 118.9 | 7.3 | 115.3 | 1.1 | 184 | 50.2 | 7.6 | 69 |
| | **5** | 127.0 | 120.5 | 6.5 | 117.0 | 2.0 | 189 | 50.9 | 15.2 | 70 |
| | **2.5** | 127.3 | 121.6 | 5.7 | 117.9 | 3.8 | 192 | 50.4 | 30.8 | 70 |
| **PE-g-2.0%** | **20** | 128.5 | 117.5 | 11.0 | 113.9 | 0.8 | 174 | 50.0 | 3.9 | 67 |
| | **10** | 128.9 | 119.3 | 9.6 | 115.9 | 1.3 | 179 | 50.3 | 7.9 | 67 |
| | **5** | 129.4 | 121.1 | 8.3 | 117.7 | 2.4 | 181 | 50.2 | 15.8 | 68 |
| | **2.5** | 129.5 | 122 | 7.8 | 118.5 | 4.6 | 182 | 50.0 | 31.9 | 69 |
| **PE-g-5.0%** | **20** | 130.1 | 118.5 | 11.6 | 115.2 | 0.9 | 169 | 50.3 | 4.0 | 65 |
| | **10** | 132.4 | 119.6 | 12.8 | 116.3 | 1.7 | 170 | 50.2 | 8.2 | 65 |
| | **5** | 134 | 121.3 | 13 | 117.8 | 3.2 | 172 | 50.3 | 16.6 | 66 |
| | **2.5** | 135.1 | 122.2 | 12.9 | 119.3 | 7.4 | 174 | 50.0 | 35.1 | 67 |

Φ is the cooling rate.

$T_0$, $T_c$, and $T_f$ denote for the onset, crystallisation, end crystallisation temperatures, respectively.

$T_{0.5}$ and $t_{0.5}$ are the temperature and time required to reach 50% of relative crystallinity, respectively.

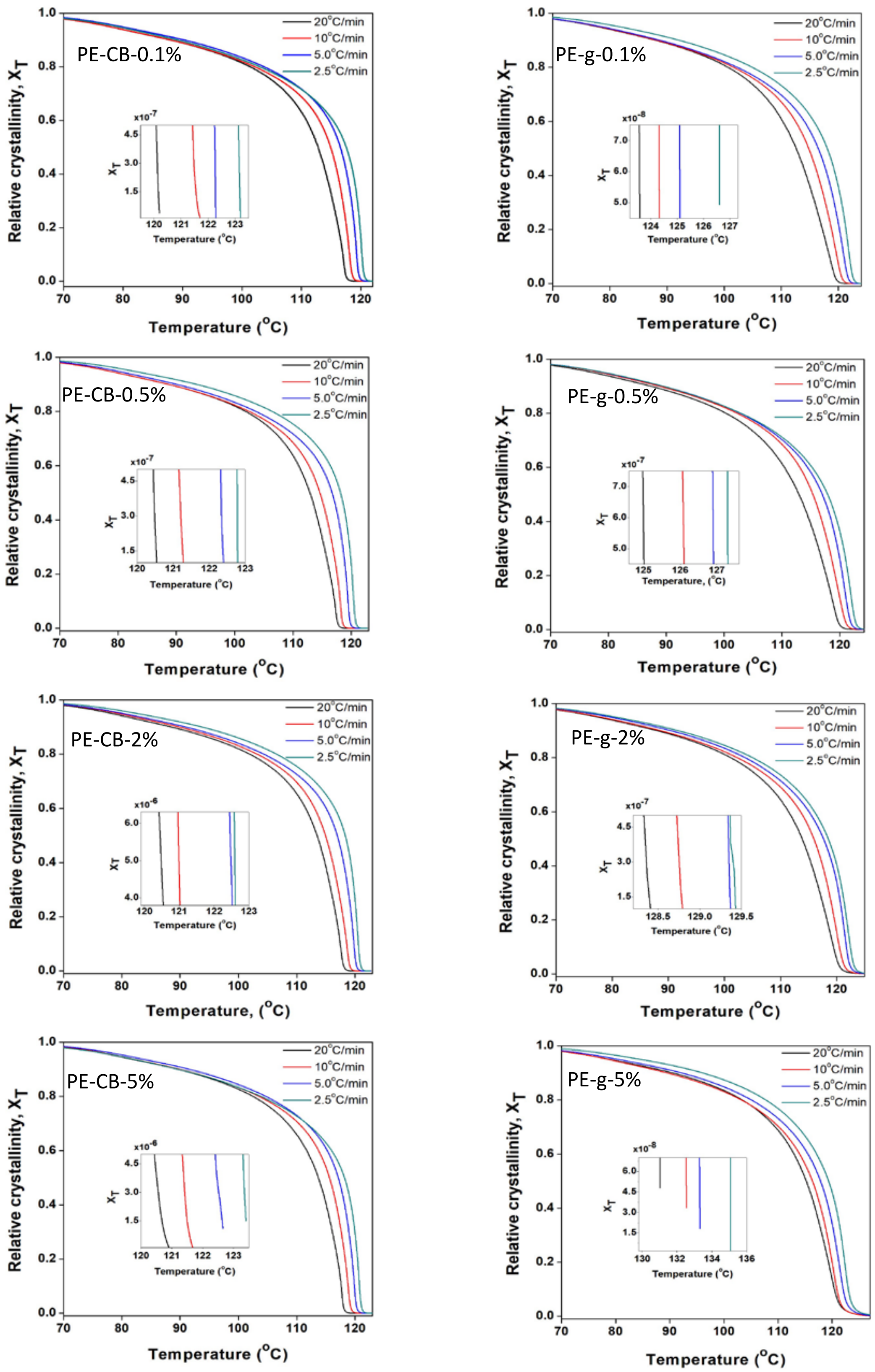


**Figure S4.** Relative crystallinity evolution as a function of crystallisation time ($X_T$) for the PE-CB, and PE-g at 0.1, 0.5, 2, 5 wt.%, occurred under non-isothermal conditions. The onset temperature of crystallisation at different cooling rates are presented in the inset.

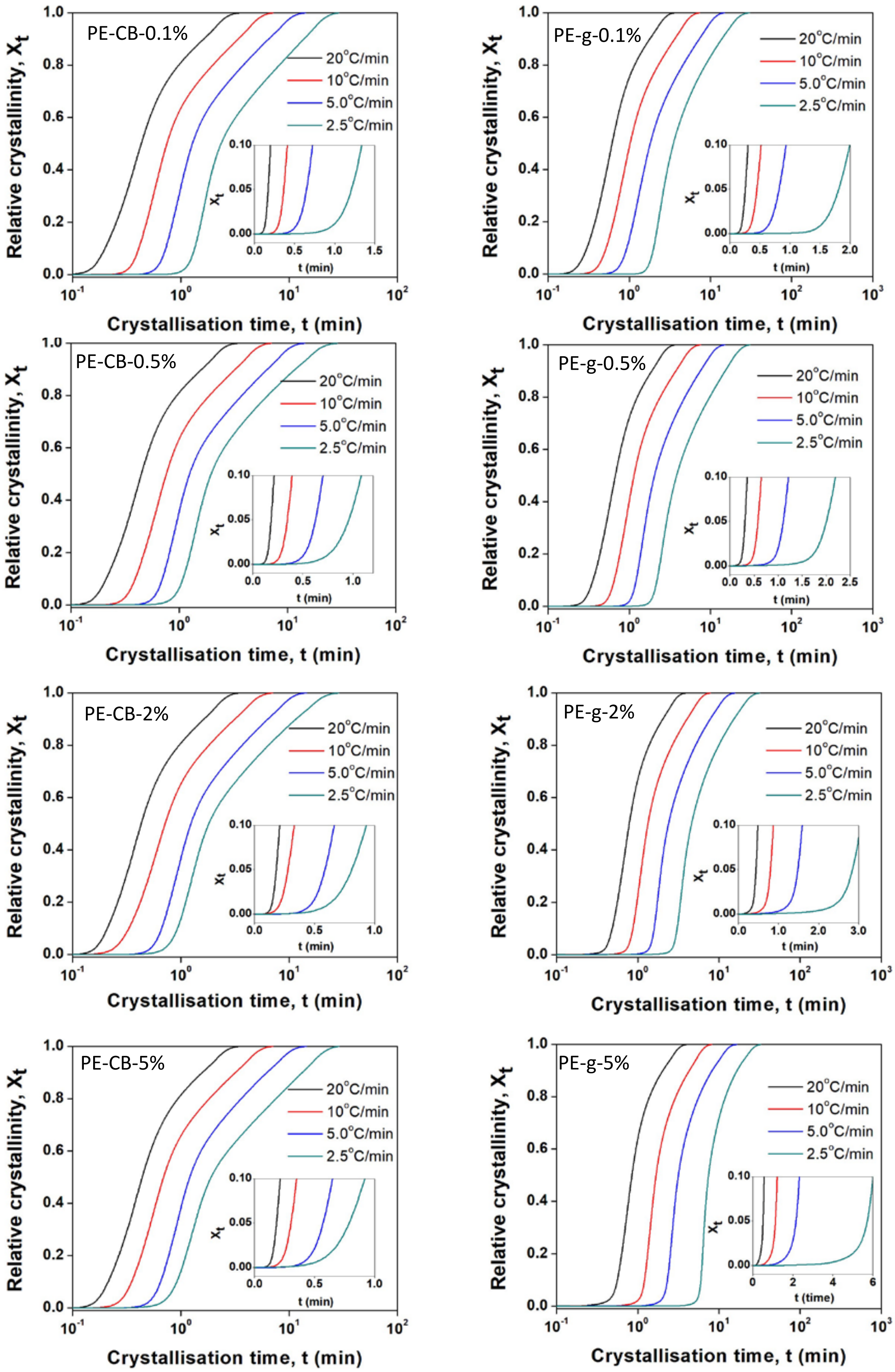


**Figure S5.** Relative crystallinity evolution as a function of crystallisation time ($X_t$) for the PE-CB, and PE-g at 0.1, 0.5, 2, 5 wt.%, occurred under non-isothermal conditions. The incubation period at different cooling rates are presented in the inset.

**Table S2.** Average molecular weights for the neat multimodal-HDPE before and after extrusion.

| Sample | Mw (g/mol) | Mn (g/mol) | Mw/Mn | Mz (g/mol) |
|---|---|---|---|---|
| **HDPE-powder** | 279663 | 8490 | 32.9 | 2098540 |
| **HDPE-Extruded** | 207077 | 8572 | 24.2 | 1131340 |

The average molecular weights were measured by Polymer-Char High Temperature Gel Permeation Chromatography (HT-GPC), which was equipped with capillary viscometer and IR5 compositional detectors, Agilent oven 7890A, two PL-gel Olexis columns (guard and analytical), using 1,2,4-trichlorobenzene as a mobile phase. The test was conducted at 140 °C with an elution speed of 1 ml/min. We could not publish the GPC figure because it is restricted by the licensing department, i.e., reasons related to the patent of the company.

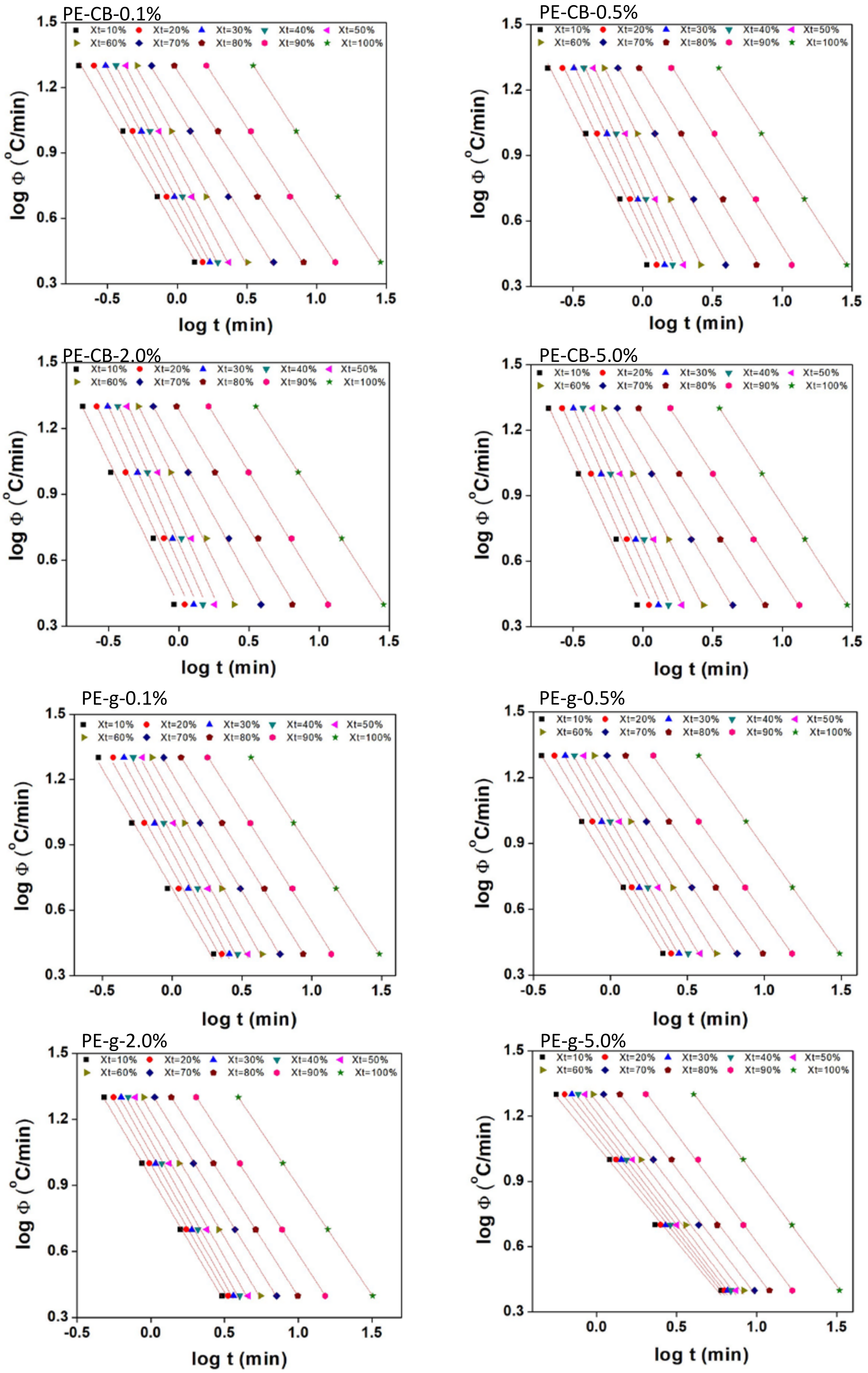


**Figure S6.** Plots of log Φ versus log *t* from the combined Avrami-Ozawa equations for the multimodal-HDPE filled with graphene or carbon black at 0.1, 0.5, 2, 5 wt.%, during the non-isothermal crystallisation.
